# Effect of ondansetron on reducing ICU mortality in patients with acute kidney injury

**DOI:** 10.1038/s41598-021-98734-x

**Published:** 2021-09-30

**Authors:** Xiaojiang Guo, Xiguang Qi, Peihao Fan, Michael Gilbert, Andrew D. La, Zeyu Liu, Richard Bertz, John A. Kellum, Yu Chen, Lirong Wang

**Affiliations:** 1 Department of Pharmaceutical Sciences, Computational Chemical Genomics Screening Center, School of Pharmacy, University of Pittsburgh, 5607 Baum Boulevard, Pittsburgh, PA 15206 USA; 2 Department of Pharmacy and Therapeutics, School of Pharmacy, University of Pittsburgh, Pittsburgh, PA 15206 USA; 3 The Center for Critical Care Nephrology Department of Critical Care Medicine, School of Medicine, University of Pittsburgh, Pittsburgh, PA 15206 USA; 4 Eli Lilly and Company, Lilly Corporate Center, Indiana, IN 46225 USA; 5 The Dietrich School of Arts & Sciences, University of Pittsburgh, Pittsburgh, PA 15206 USA

**Keywords:** Computational biology and bioinformatics, Drug discovery, Systems biology

## Abstract

The purpose of this study is to identify medications with potentially beneficial effects on decreasing mortality in patients with acute kidney injury (AKI) while in the intensive care unit (ICU). We used logistic regression to investigate associations between medications received and ICU mortality in patients with AKI in the MIMIC III database. Drugs associated with reduced mortality were then validated using the eICU database. Propensity score matching (PSM) was used for matching the patients’ baseline severity of illness followed by a chi-square test to calculate the significance of drug use and mortality. Finally, we examined gene expression signatures to explore the drug’s molecular mechanism on AKI. While several drugs demonstrated potential beneficial effects on reducing mortality, most were used for potentially fatal illnesses (e.g. antibiotics, cardiac medications). One exception was found, ondansetron, a drug without previously identified life-saving effects, has correlation with lower mortality among AKI patients. This association was confirmed in a subsequent analysis using the eICU database. Based on the comparison of gene expression signatures, the presumed therapeutic effect of ondansetron may be elicited through the NF-KB pathway and JAK-STAT pathway. Our findings provide real-world evidence to support clinical trials of ondansetron for treatment of AKI.

## Introduction

Acute kidney injury (AKI) is an abrupt and usually reversible decline in glomerular filtration^1^, which is attributed to various causes^2,3^, is a common disorder and is encountered in various clinical settings^4,5^. Nearly 60% of patients worldwide will suffer from AKI during their intensive care unit (ICU) stay^6^. AKI is usually unavoidable because of the ineffective preventive therapies. Furthermore, patients often already have undergoing AKI when they receive medical attention. Even when AKI develops in the hospital, recognition is often delayed so treatment is the only option^7^. AKI results in a 1-year mortality of 20–50% in critically ill patients^8^. Patients with AKI also have poor short-term prognoses such as prolonged ICU and hospitalization stays and significantly reduced hospital survival. Future development of chronic kidney disease, and risk for chronic dialysis and/or kidney transplantation are also known sequelae^9^. Unfortunately, the pathobiology of AKI is still unclear and no drugs have yet been approved specifically targets AKI^10^.

Therapy for critically ill patients with AKI requires coordination of a number of treatments across multiple disciplines^11^. In 2012, Kidney Disease: Improving Global Outcomes (KDIGO) published the first interdisciplinary and international clinical practice guideline on AKI^4^. Recommendations were provided for supportive care, but no specific therapies were recommended.

Multiple comorbidities of AKI have been shown by epidemiology studies including cancer^12^, cardiovascular diseases^13^, complex surgery^14^, liver diseases^15,16^, diabetes mellitus^17^, and subsequent infection/sepsis^18,19^. Given the range of surgical and medical conditions associated with AKI, multiple medications are prescribed. In this study, we analyzed two publicly available databases (MIMIC III and eICU) of electronic medical records (EMR) from more than 20,000 patients who incurred one or more episodes of AKI during an ICU stay. Our focus was to determine associations between medication use and ICU mortality. Through this analysis, we aimed to identify drugs already used in clinical practice for treating diseases other than AKI with potential beneficial effects for AKI.

## Results

### Variables associated with ICU mortality in patients with AKI

From the Multiparameter Intelligent Monitoring in Intensive Care III (MIMIC III) database, we identified 9,536 patients with AKI, of whom 9,443 had completed information on demographics, ICU stay, and the first day vital information. We further excluded those patients with multiple ICU stays to simplify the calculation, resulting in 7,313 unique patients. We also excluded 286 patients with diagnosis codes of 585.5 or 585.6, and finally got 7,027 AKI patients. The basic characteristic of patients in the MIMIC-III cohort can be found in Appendix A. We used a Chi-Squared Test for categorical variables and t test for continuous variables. We used information from these 7,027 patients to build a logistic regression model and identified factors that contributed significantly to the prediction of ICU mortality (95% CI). Table 1 lists variables with *p* values < 0.05. The accuracy, and AUC (area under curve) of this logistic regression model are 0.838and 0.843 respectively. Estimate coefficients show two sides of effect, positive and negative. A negative coefficient means that a factor is associated with increased survival vice versa. Death rates among patients with AKI who had ever used the top 50 drugs can be found in Appendix B.

**Table 1:** Estimated coefficients and significance of factors that influence ICU mortality of patients with AKI.

Variables	Coefficient	95% Confidence interval		Pr( >|Z|)
Warfarin	− 0.115	− 0.139	− 0.090	2.971E−20
Oxycodone	− 0.089	− 0.110	− 0.068	2.456E−16
Haloperidol	− 0.063	− 0.086	− 0.039	2.224E−07
Magnesium Sulfate	− 0.054	− 0.071	− 0.037	8.970E−10
Heparin	− 0.053	− 0.071	− 0.036	3.328E−09
Metoprolol	− 0.051	− 0.070	− 0.033	3.653E−08
Glucagon	− 0.050	− 0.074	− 0.026	4.959E−05
Lisinopril	− 0.049	− 0.075	− 0.024	1.323E−04
Captopril	− 0.044	− 0.076	− 0.011	0.008
Acetaminophen	− 0.040	− 0.058	− 0.023	5.134E−06
Furosemide	− 0.036	− 0.054	− 0.018	8.060E−05
Ondansetron	− 0.035	− 0.056	− 0.014	0.001
Hydralazine	− 0.035	− 0.058	− 0.011	0.004
Docusate	− 0.028	− 0.050	− 0.006	0.012
Pantoprazole	− 0.025	− 0.042	− 0.008	0.004
Hydromorphone	− 0.024	− 0.048	− 0.001	0.042
Bisacodyl	− 0.021	− 0.041	− 0.001	0.043
Creatinine average	− 0.020	− 0.027	− 0.013	5.915E−08
SpO2 mean	− 0.006	− 0.009	− 0.004	8.278E−09
Platelet average	− 1.165E−04	− 1.870E−04	− 4.603E−05	0.001
BUN average	4.633E−04	5.097E−05	0.001	0.028
PTT average	0.001	0.001	0.002	3.457E−13
Bilirubin average	0.002	0.000	0.004	0.037
Resprate mean	0.005	0.003	0.007	1.840E−07
Sapsii	0.006	0.005	0.007	1.184E−44
Anion gap average	0.011	0.004	0.018	0.003
Lactate average	0.016	0.011	0.021	1.400E−09
Potassium average	0.020	0.006	0.035	0.007
Midazolam	0.027	0.001	0.054	0.043
Lorazepam	0.031	0.014	0.049	0.001
Stroke	0.066	0.029	0.103	4.373E−04
Fentanyl	0.076	0.050	0.102	8.943E−09
Meropenem	0.076	0.047	0.106	4.689E−07
Solid tumor	0.078	0.017	0.140	0.012
Amiodarone	0.090	0.065	0.115	2.719E−12
Norepinephrine	0.167	0.144	0.190	6.629E−46
Morphine	0.198	0.181	0.215	7.624E−109
Coagulopathy	0.809	0.186	1.431	0.011

SAPSII: Simplified Acute Physiology Score (SAPS) II; PTT: Partial thromboplastin time; SpO2: Oxygen saturation; KDIGO: Kidney Disease Improving Global Outcomes; Bun: blood urea nitrogen.

### Drugs with potentially beneficial effects on AKI mortality

We performed a literature search on all drugs identified to have effects of mortality (Appendix C). Nine of 22 drugs with negative coefficients in Table 1 were reported to have a beneficial effect on decreasing ICU mortality. Among all drugs, ondansetron stood out as an antiemetic, which seemed to be less related on preventing death and AKI recovery. Ondansetron showed the best performance on decreasing ICU mortality in patients with AKI with the least connection of indication on a previously identified life-saving effect.

### Validation of beneficial effects of ondansetron using the eICU database

We identified 14,338 patients with AKI from the eICU database. 5439 patients were excluded because of missing data or having multiple ICU stays. And 192 patients with ICD9-CM codes of 585.5 or 585.6 were also excluded. Finally, we got 11,041 AKI patients from eICU database. The mean baseline characters values of patients’ receiving/not receiving ondansetron were significantly different. We applied a 1:1 propensity score matching (3423:3423 patients were matched) to balance the patients’ physiology condition between treatment and control group. Detailed basic characteristics of eICU patients before and after matching can be found in Appendix D and E.

After the baseline adjustment by propensity score matching, we saw a significantly lower ICU mortality (12.56%) in the ondansetron group than in the non-ondansetron matched control group (15.16%), *p* = 0.002085 (Table 2). Death rates among patients with AKI with the 50 most frequently used drugs from MIMIC III and eICU can be found in Appendix B.

**Table 2:** The contingency table of the death events occurred in patients receiving/not receiving ondansetron. P-value is calculated with chi-square test.

	Non-Ondansetron	Ondansetron	Total	*p* value
Death	519	430	949	0.002085
Alive	2904	2993	5897	
Total	3423	3423	6846	

### Molecular mechanism study by gene expression signatures

**Figure 1:**
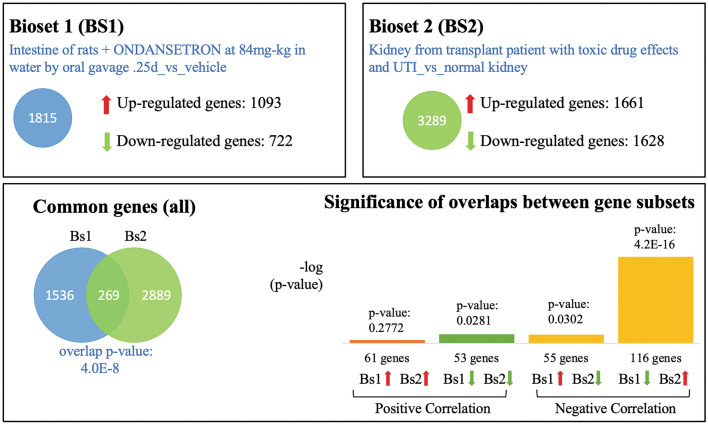
Detailed comparison between ondansetron-induced gene expression profile and kidney from transplant patients with toxic drug effects.

We further analyzed the gene expression profiles induced by ondansetron and AKI. Through the BaseSpace database, we found one ondansetron dataset from the Chemical Effects in Biological Systems database^20^, where intestines from rats were treated with ondansetron in vivo and assayed for expression. We selected the intestine of rats + ondansetron at 84 mg-kg in water by oral gavage 0.25d vs. vehicle to mimic the acute effects of this drug. Searching with this gene expression profile, we found three biosets^21^ of AKI from transplant patients with toxic drug effects (biosets 2, 3 and 4 in Table 3). They overlapped with the ondansetron bioset (bioset 1 in Table 3) with p values of 4.0E−08, 9.8E−07 and 4.9E−06, respectively. A detailed comparison is shown in Fig. 1. We can see that ondansetron and AKI (bioset 2) have 1,815 and 3,289 differentially expressed genes (DEGs), respectively, and they shared 269 common DEGs with significant p-values of 4.0E−8. Among those overlapping DEGs, 61 were both upregulated and 53 were both downregulated. A total of 55 genes were upregulated by ondansetron but were downregulated by AKI, while 116 genes were downregulated by ondansetron but were upregulated by AKI in bioset 2.

**Table 3:** Comparation of ondansetron-induced gene expression profiles with three AKI gene expression profiles.

Biosets	Bioset Name	Genes	Overlap *p* value	Common Genes
Bioset 1 (Ondansetron)	Intestine of rats + ONDANSETRON at 84 mg-kg in water by oral gavage 0.25d _vs_ vehicle (GEO ID: GSE59927)	1815	–	–
Bioset 2(AKI)	Kidney from transplant patient with toxic drug effects and UTI _vs_ normal kidney (GEO ID: GSE362)	3289	4.0E−08	269
Bioset 3 (AKI)	Kidney from transplant patient with toxic drug effects _vs_ normal kidney (GEO ID: GSE409)	4417	9.8E−07	345
Bioset 4(AKI)	Kidney from transplant patient with toxic drug effects, UTI, and ARII_vs_ normal kidney (GEO ID: GSE410)	4695	4.9E−06	345

UTI: urinary tract infection, ARII: acute rejection type II.

### Validation of ondansetron gene signature in the transcriptome from a pure AKI cohort

**Figure 2:**
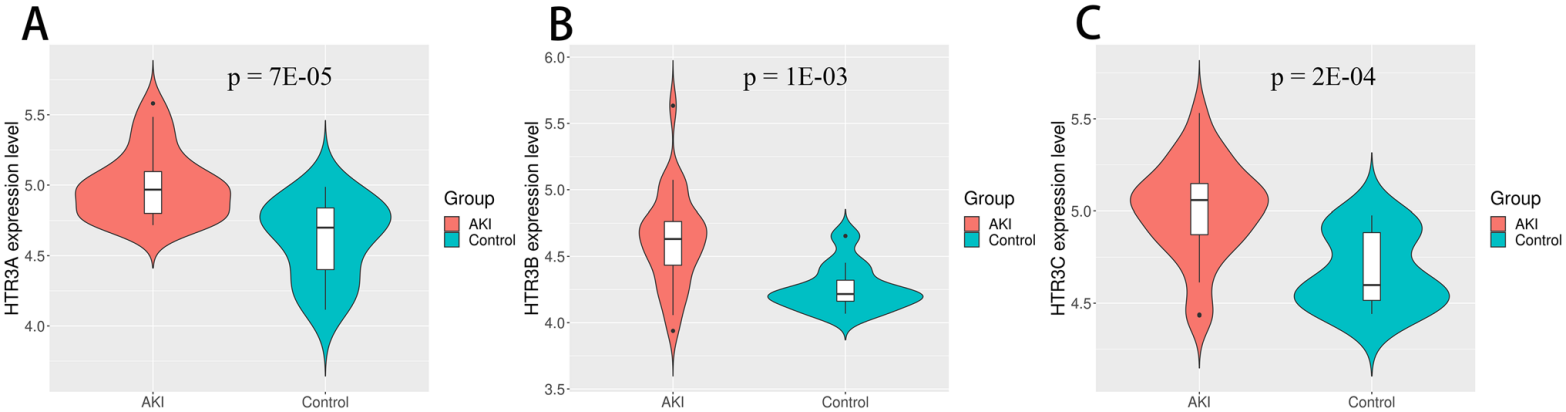
Changes of HT3 receptor genes (HTR3A, HTR3B and HTR3C) in patients with AKI compared with control (the pristine protocol biopsies).

**Figure 3:**
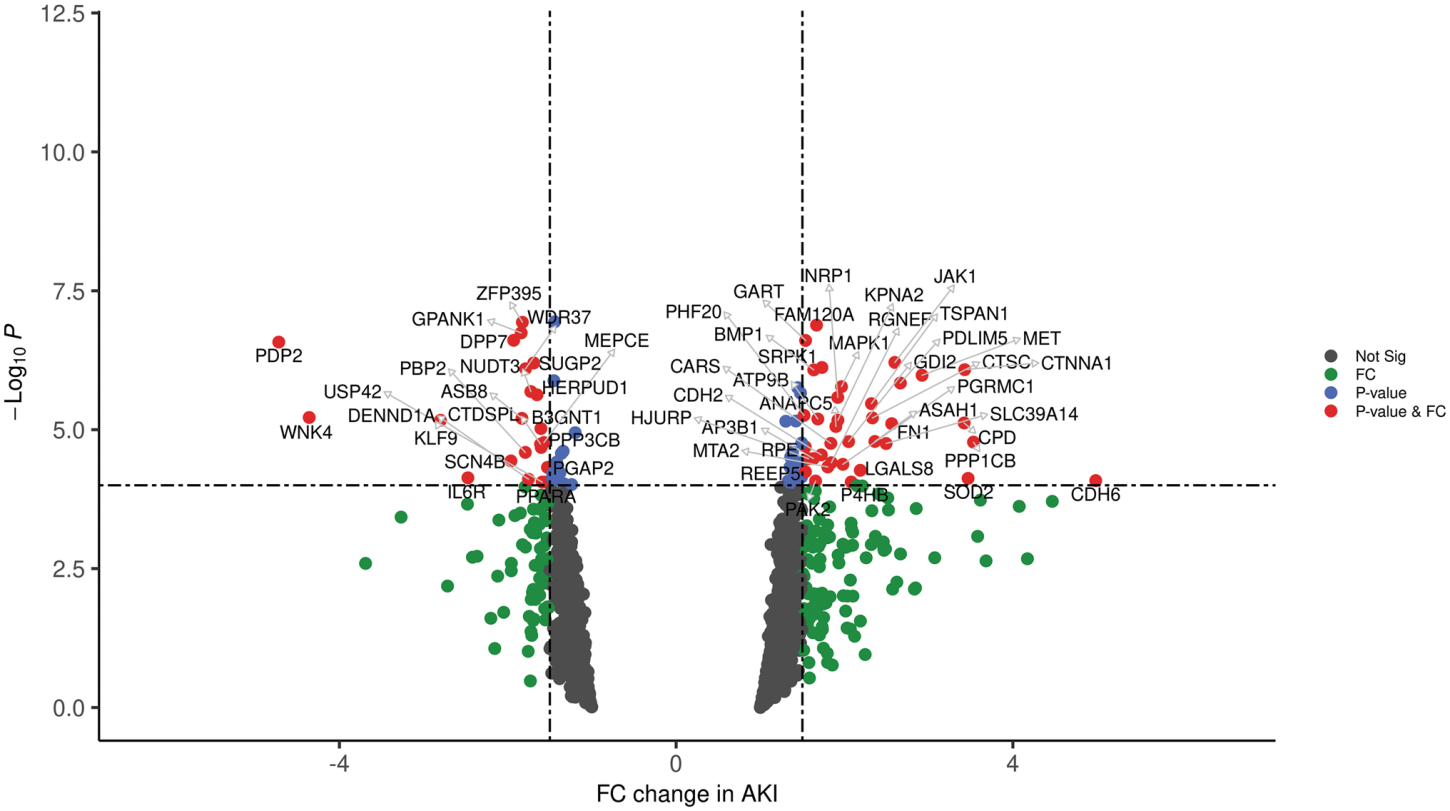
Volcano plot of gene expression changes of AKI compared with control.

Given that ondansetron is a 5-HT3 receptor antagonist, we examined the transcriptomes of 5-HT3 receptor genes (HTR3A, HTR3B and HTR3C). By neutralizing the ubiquitous minor changes inevitably induced by the kidney transplant process, the comparison of AKI kidneys to histologically pristine protocol biopsies of stable transplants will reveal the molecular features of AKI. In this transcriptome study, 5-HT3 receptor genes were all shown to be significantly upregulated (Fig. 2). A volcano plot of the comparison results between the AKI and pristine protocol biopsy demonstrated significant positive and negative gene changes among the Ondansetron bioset. The volcano plot showed that JAK1, MAPK1, CTNNA1 and MET were upregulated in AKI by both fold change and P-values (Fig. 3). Of note, Rela is not in the upregulated gene list because the fold change was 1.49, which is around the threshold. Conversely, FN1, which displayed little change in the other three biosets above, changed significantly. FN1 was reported to be associated with AKI by the comparative toxicogenomics database^22^ with 499 references. Among the top 30 genes that were inversely correlated with estimated glomerular filtration rate (eGFR) at the time of biopsy in AKI biopsies, 8 genes were transcriptionally modulated by ondansetron. All 8 genes were upregulated in the human AKI cohort, and interestedly, we observed 6 out of 8 genes whose gene expression was downregulated by Ondansetron (Table 4). Finally, we examined genome-wide expression changes of all ondansetron pharmacological signature genes in this cohort. The number of differentially expressed genes (AKI vs control) was significantly enriched with a P value of 2.2E−11 (the Appendix table Ondansteron_geneSignature_inhumanAKIgenomicsdata.docx).

**Table 4:** Genes whose expressions were negatively correlated with eGFR were also modulated by Ondansetron.

Gene Symbol	Gene title	Correlation of gene mRNA expression with eGFR in human AKI cohort	Gene expression mRNA change in GSE30718 (AKI vs normal control)		Gene expression fold change by Ondansetron treatment
			*p* value	Fold change	
KPNA2	Karyopherin a2 (RAG cohort 1, importin a1)	− 0.72	6.73E−06	1.9	1.68
CASP1	Caspase 1, apoptosis-related cysteine				
peptidase (IL-1, b, convertase)	− 0.72	1.00E−03	2.1	− 8.03	
TFPI	Tissue factor pathway inhibitor (lipoproteinassociated				
coagulation inhibitor)	− 0.7	0.01	1.5	4.25	
AMACR	a-methylacyl-CoA racemase	− 0.68	2.80E−04	2.3	− 2.38
GBP2	Guanylate binding protein 2, IFN-inducible	− 0.67	1.00E−03	2.1	− 4.71
MCL1	Myeloid cell leukemia sequence 1 (BCL2-related)	− 0.66	3.90E−05	2.8	− 3.07
MET	Met proto-oncogene (hepatocyte growth factor receptor)	− 0.66	1.05E−06	2.9	− 2.32
CPD	Carboxypeptidase D	− 0.66	7.50E−06	3.4	− 2.4

## Discussion

Most previous EMR-based studies have focused on nephrotoxic effects of medications^23–25^. There is limited prior literature that uses EMRs to repurpose FDA approved drugs for AKI treatment. The development of new drugs is a costly endeavor with an average cost approaching a billion dollars^26^, and the time for a new drug from preclinical compound to marketing can take up to 20 years^27^. Costs and time can be greatly reduced through repurposing^28^. From a practical point of view, a drug identified from our study can be a potential treatment option for AKI patients with a validated safety profile since it has been safely used in AKI patients for other indications. However, more research is needed both to establish the efficacy of ondansetron for AKI treatment as well as to determine optimal dosing and duration of therapy.

Among the drugs we found to be associated with reduced mortality in critically ill patients with AKI, ondansetron is of most interest for several reasons. First, it is an antiemetic drug that there are no reports or clinical trials to suggest its beneficial effect on decreasing mortality either in patients with AKI or other ICU patients. Second, ondansetron is a selective antagonist on the serotonin (5-HT3) receptor^29^, which is a receptor with wide distribution in the human body^30^ and its expression is upregulated in AKI (Fig. 3). This might suggest that this receptor is a drug target in AKI.

We recognize that the lower mortality in the ondansetron-treated group might be due to indication bias. Ondansetron is indicated for nausea which only awake, communicative patients can report. Patients in the ondansetron group thus may have lower disease severity. We controlled for this possibility by matching propensity score of ondansetron users with non-users and still found a significant, albeit smaller, impact on mortality in this matched analysis. However, in the analysis of eICU patients, the Number Need to Treat (NNT) was 38.46 [95% confidence interval 23.61, 103.72]. This relatively high NNT may be due to the fact that patients were not prescribed ondansetron for treatment of AKI but for prevention/treatment of nausea and vomiting. These patients received comparably little dose in an irregular way. For example, when in the eICU database, there were 6,685 records of ondansetron use for 3,848 patients. Among these records, 5,849 (87.5%) were labeled as “prn”(as needed). For route of administration, 692 records were oral and 5,729 records were labeled as injections.

We further explored the possible molecular mechanisms by comparing gene expression signatures. Generally, if the genes regulated by the drug and the disease are in the opposite direction but show a significant overlap, the drug may have a potential therapeutic effect on the disease. Our analysis revealed that there was a remarkable overlap of genes affected by both ondansetron and AKI. The most significant overlap occurred on genes that are upregulated by AKI but downregulated by ondansetron. Furthermore, by validating the ondansetron gene signature in the “pure AKI” cohort, 5-HT3 receptor genes were significantly upregulated in patients with AKI. Hence the potential beneficial effect of ondansetron on AKI has support from molecular mechanisms.

To investigate the possible molecular mechanism of ondansetron on modulating AKI-related molecular pathways, we performed a meta-analysis on four biosets with the integrated function of BaseSpace. We found that pathways in cancer, microRNA target genes by miR381, miR200b, miR101, and miR26, were downregulated (Appendix F). Furthermore, miR381 was reported to play a role in rat models of renal ischemia reperfusion injury^31^. Target genes miR200b, miR101, and miR26 can be used as biomarkers for AKI^32,33^. As we can see from Appendix G, among the downregulated genes, Rela and Jak1 are the key proteins in the NF-KB pathway and JAK-STAT pathway, respectively. In comparison, these two genes are upregulated in AKI. Inhibitors for these two pathways have been reported to have beneficial effects for AKI^34,35^.

There are limitations to this study. In our analysis, we adjusted for other medications and comorbidities to mitigate the effects of confounders in logistic regression, and we used matching to help alleviate the possible bias in baseline severity of illness in the validation step. However, we still cannot rule out the possibility of unknown confounder effects. In addition, ondansetron is approved in cancer patients and in the post-operative setting, implying the possibility of indication bias. However, a recent study examining drug combinations reported that ondansetron may *increase* the risk of AKI^36^. In patients receiving chemotherapy, GI symptoms may correlate with nephrotoxicity and thus it is difficult to say if an anti-emetic is a marker or mediator of AKI. In any case, these data do not support indication bias as an explanation for our findings. Finally, from gene expression data of molecular mechanism analysis, we have confidence that ondansetron has an effect on pathways relevant to AKI. As such, we believe that our results provide evidence that further study is warranted.

## Materials and methods

### Research design

**Figure 4:**
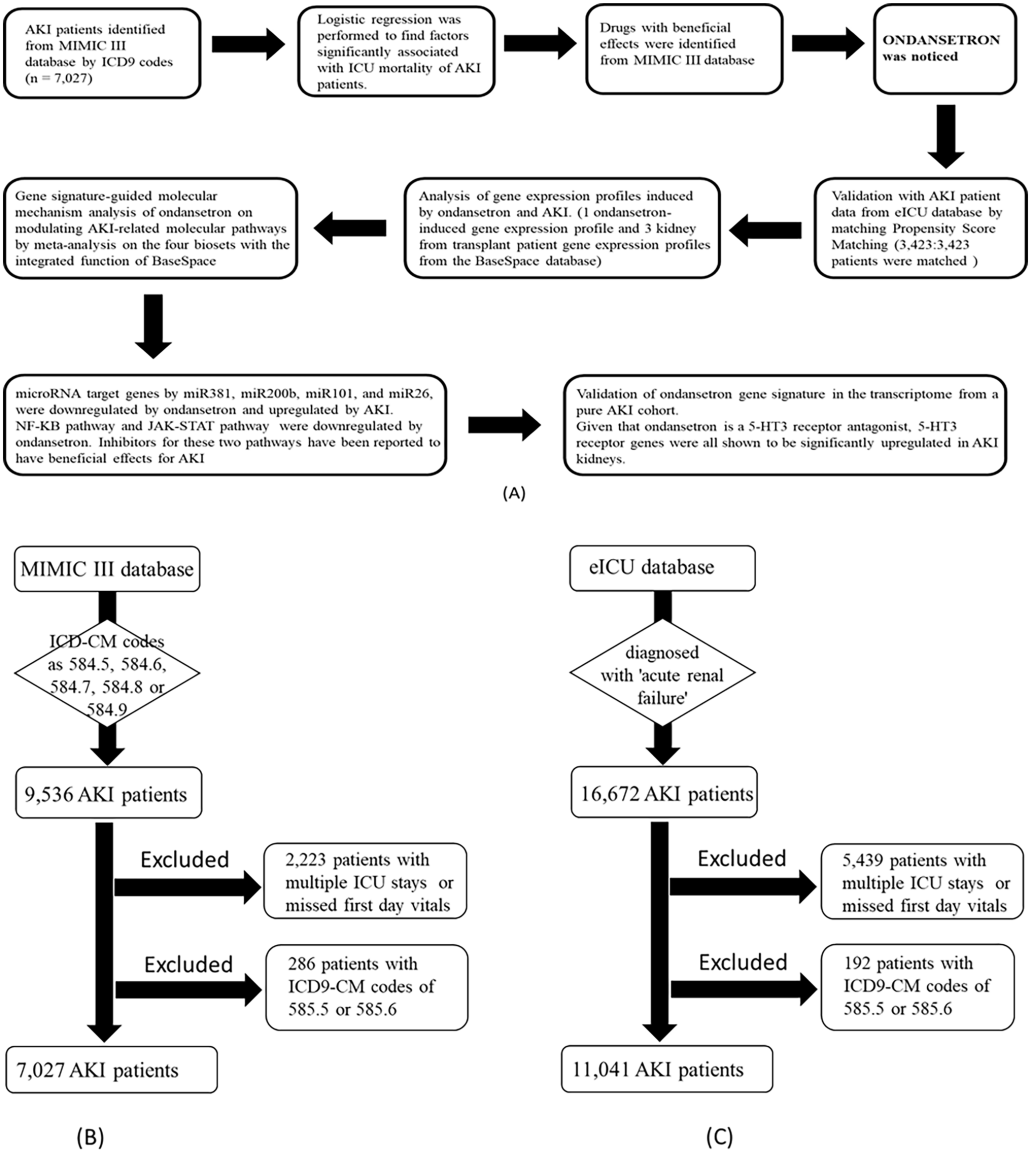
The flowcharts of procedures (**A**) The overview of the experimental design. (**B**) The flowchart of AKI patient inclusion in MIMIC III database. And (**C**) the flowchart of AKI patient inclusion in eICU database. AKI: acute kidney failure.

The flowcharts of our procedures are shown in Fig. 4. We first identified patients with AKI from the MIMIC III database by using International Classification of Diseases, Ninth Revision (ICD9) codes. Clinical data was extracted (age, gender, medication use information and other variables to determine patients’ conditions). We used logistic regression to select variables that showed significant beneficial effects on ICU mortality. Among these variables, we identified drug(s) with potential beneficial effects and conducted literature searches to confirm the plausibility. We then used data from a second independent database (eICU) to validate our findings, and we focused on drugs that were not expected to have a direct effect on survival from their primary use (e.g. drugs used to manage symptoms but not life-threatening conditions). Finally, we used gene signature analysis to find possible mechanisms for each drug candidate for their beneficial effects on AKI.

### Data source

Our discovery phase utilized data from the MIMIC III v1.4, which is the latest version of an openly available clinical database developed by the MIT Lab for Computational Physiology^37^. This database comprises more than 60,000 ICU admissions to the Beth Israel Medical Center, Boston MA, from June 2001 to October 2012, including patient demographics, past medical history, laboratory tests, medication records, and diagnoses. To acquire access to MIMIC III, we completed the CITI “Data or Specimens Only Research” course (record ID: 36580723). ICD9-CM codes, laboratory test results, medications and time events were extracted. The project was approved by the Institutional Review Boards of Beth Israel Deaconess Medical Center and the Massachusetts Institute of Technology (Cambridge, MA). To guarantee privacy of patients, data were deidentified. Informed consent was not required, as only retrospective deidentified data from public databases was used.

We used a second database, eICU, to validate our findings from the discovery phase^38^. The eICU database is a collaborative research database that consists of over 200,000 multi-center critical care records in ICUs in the United States through 2014–2015 and was made available by Philips Healthcare in partnership with the MIT Laboratory for Computational Physiology. The CITI “Data or Specimens Only Research” course was also required for access to this database. Data were deidentified to safeguard patient privacy.

We used the Illumina BaseSpace software to explore possible molecular mechanisms of drugs that have a potentially beneficial effect in AKI^39^. The BaseSpace software consists of a number of apps that provide next-generation sequencing, transcriptional, and proteomic data analysis mostly developed or optimized by Illumina.

### Population selection criteria

We used the ICD9-CM codes 584.5, 584.6, 584.7, 584.8 and 584.9 to search the diagnosis table in the MIMIC III database to identify patients with AKI. For eICU database, we used the keyword search (‘acute renal failure’) in the diagnosis table to identify patients with AKI. We further excluded those patients with multiple ICU stays or with first day vitals missing to simplify the calculation. We also excluded patients with ICD9-CM codes of 585.5 (Chronic kidney disease, Stage V) and 585.6 (End stage renal disease) (Fig. 4B,C). Because the focus of this study is mortality in ICU stays for AKI patients, no censor strategies were applied. In other words, upon discharging from ICU units, the patients were either alive or dead.

### Data extraction and missing values process

From the MIMIC III database, we extracted the demographic characteristics, physiological index, ICD9 codes, medications, laboratory tests, and vital status (alive or dead) upon ICU discharge. These variables were classified into three categories: first day vital information, medication use information, and other variables (Appendix H). Because Ondansetron is used for nausea and vomiting, especially in post-surgical patients, we also consider the surgical status of those patients. For MIMIC3 database, we searched the CPTEVENTS table on the SECTIONHEADER containing “Surgery”, while for eICU database, we searched the diagnosis table on the diagnosisstring containing “surgery”.Medication use information was filtered to be the top 50 most used drugs among these patients with AKI. From the eICU database, we extracted physiology characters, comorbidities situation, medications, and vital status on ICU discharge. The codes for comorbidities were from GitHub (Appendix I). The codes for first day labs and vital information were also from GitHub(https://github.com/MIT-LCP/mimic-code/tree/main/mimic-iii/concepts/firstday). Missing values were found in physiological indexes such as average heart rate, average systolic blood pressure, average blood glucose and average albumin counts. For MIMIC III data, all variables containing missing values were continuous variables, thus we filled the missing values with means of the whole column^40^.We extracted drug and disease induced DEGs by using the Illumina BaseSpace software.

### Logistic regression to identify variables that significantly influence ICU mortality of patients with AKI

We further analyzed the extracted data using multivariate logistic regression. Death within 24 h of ICU discharge was considered as the primary outcome. Eleven variables were dropped because of collinearity between covariates for logistic regression, including paralysis, hypothyroidism, peptic ulcer disease, obesity, weight loss, blood loss anemia, deficiency anemias, drug abuse, psychoses, cardiac arrhythmia, and depression.

### Validation of our findings using the eICU database

We then further validated our findings with the eICU database. Patients’ physiology characters (temperature, respiratory rate, heartrate, mean blood pressure and creatinine), surgical status, and their comorbidities situation (Including comorbidities that affect larger than 5% patients in either treatment or non-treatment group) were used for propensity score matching^41^ of the patient’s baseline severity of illness followed by a chi-square test to calculate the significance of drug use and mortality. Propensity score matching process was conducted using R package Match It function “matchit”^42^ (method = "nearest", ratio = 1, discard = "both", caliper = 0.05). Our assumption is that those matched patients will have similar physiology condition. If a drug has beneficial effect on AKI, the death rate of the user group will be lower than that of the non-user group.

### Investigation of possible molecular mechanisms by comparison of gene transcriptional profiles

To understand the molecular mechanism behind the potential beneficial effects of a drug of interest on AKI mortality, we analyzed the gene expression profiles induced by drugs associated with lower ICU mortality in our analyses. DEGs induced by drugs were collected from Illumina BaseSpace software. In BaseSpace, only genes with *p* values < 0.05 and absolute fold changes greater than 1.2 were considered as DEGs. All the DEGs induced by a drug can be considered as a gene signature for this drug. The drug-induced gene expression datasets were selected by searching with drug names. We then used the drug-induced DEGs to search against disease-induced DEGs (three biosets, names can be found in Table 3) to find potential associations between these drugs and kidney diseases through the commonly modulated genes. The molecular pathways of those common genes involved were collected through meta-analysis function in BaseSpace to investigate possible molecular mechanisms for beneficial effects. All BaseSpace analyses were performed using the default parameters.

### Validation of ondansetron gene signature in a human “pure AKI” cohort

To further elucidate the molecular mechanisms of ondansetron in AKI, we validated the gene signature of ondansetron in transcriptome data from a pure AKI cohort (GEO ID: GSE30718). Because some degree of AKI happens in all kidney transplantation patients, an excellent human AKI model can be found in early kidney transplants without rejection. In a prospective study of 234 kidney transplant biopsies for clinical indications, kidneys with rejection and kidney disease (other than AKI) by histologic criteria were excluded, and those with nondiagnostic suspicious histologic lesions were also excluded^43^.

These criteria identified a “pure AKI” cohort of 28 biopsies with a mean age of 52 (16–75), 15 (57.6%) living donors and with mean eGFR of 26 ml/min^43^. A total of 11 pristine protocol biopsies represented kidneys with a stable future function (at least 2 years of follow-up) after transplantation, no evidence for AKI or rejection by histology, and no clinical indication for biopsy (clinical or subclinical, before or after biopsy) were used as the controls in this study. The statistical comparison was obtained by estimated marginal means (also known as least-squares means) using R^44^. Through orthology mapping, we were able to identify 1,333 gene expression alternations in the human AKI cohort. The DEG were defined using a stringent threshold of 1.5-fold changes and a p value of less than 0.0001.

### Statistical analysis

The logistic regression was performed in R using caret package to measure the association between patient death and variables. The χ2 (chi-square) test was performed to evaluate the difference of death rates between target medication users and non-users. Matching process to balance the propensity score of the patient of both groups was conducted using R package Match It^42^ function “matchit” (method = "nearest", ratio = 1, discard = "none", caliper = 0.05). Student t-test^45^ was used to measure the mean difference between the two samples. All statistical t-tests were calculated by R. The Running Fisher algorithm is used by BaseSpace software to assess the statistical significance of overlapping between two gene sets, where p-values are computed by a Fisher’s exact test^46^. A *p* < 0.05 was used as the threshold for statistical significance for all analyses except where stated otherwise.

## Conclusions

We identified a number of candidate drugs with apparent beneficial effects to reduce ICU mortality in patients with AKI. Ondansetron, a drug that has never been reported before as a treatment for AKI, is proposed to be associated with improved survival following AKI in two independent databases. In addition, ondansetron can downregulate AKI-related genes and genes expressed through 5-HT3 receptor activation (and hence targeted by ondansetron) are upregulated in patients with AKI. Our findings provide real-world evidence to support the need for clinical trials of ondansetron to treat AKI.

## Supplementary Information

Supplementary Information.

## Data Availability

Full gene lists used in this study can be found in the supplementary material, more detailed data are available on request.

## References

[1] James MT (2010). Glomerular filtration rate, proteinuria, and the incidence and consequences of acute kidney injury: A cohort study. Lancet.

[2] Basile DP, Anderson MD, Sutton TA (2011). Pathophysiology of acute kidney injury. Compr. Physiol..

[3] Makris K, Spanou L (2016). Acute kidney injury: Definition, pathophysiology and clinical phenotypes. Clin. Biochem. Rev..

[4] Kellum, J.A., et al., *Kidney Disease: Improving Global Outcomes (KDIGO) Acute Kidney Injury Work Group. KDIGO Clinical Practice Guideline for Acute Kidney Injury.* Kidney international supplements, 2012. **2**(1): p. 1–138.

[5] Kellum, J.A., N. Lameire, and K.A.G.W. Group (2013). Diagnosis, evaluation, and management of acute kidney injury: A KDIGO summary (Part 1). Crit. Care.

[6] Hoste EA (2006). RIFLE criteria for acute kidney injury are associated with hospital mortality in critically ill patients: A cohort analysis. Crit. Care.

[7] Legrand, M. and M. Darmon, *Biomarkers for AKI Improve Clinical Practice: Yes*. 2015, Springer. 10.1007/s00134-014-3530-2 25387817

[8] Luo M (2017). A new scoring model for the prediction of mortality in patients with acute kidney injury. Sci. Rep..

[9] Schetz M (2015). Recovery from AKI in the critically ill: Potential confounders in the evaluation. Intensive Care Med..

[10] Bellomo R, Kellum JA, Ronco C (2012). Acute kidney injury. Lancet.

[11] Wekerle T (2017). Strategies for long-term preservation of kidney graft function. Lancet.

[12] Christiansen CF (2011). Incidence of acute kidney injury in cancer patients: A Danish population-based cohort study. Eur. J. Intern. Med..

[13] Fang Y (2010). Acute kidney injury in a Chinese hospitalized population. Blood Purif..

[14] Morgan CJ (2013). Risk factors for and outcomes of acute kidney injury in neonates undergoing complex cardiac surgery. J. Pediatrics.

[15] Barri YM (2009). Acute kidney injury following liver transplantation: Definition and outcome. Liver Transpl..

[16] Garcia-Tsao G, Parikh CR, Viola A (2008). Acute kidney injury in cirrhosis. Hepatology.

[17] Thakar CV (2011). Acute kidney injury episodes and chronic kidney disease risk in diabetes mellitus. Clin. J. Am. Soc. Nephrol..

[18] SooHoo M (2018). Acute kidney injury is associated with subsequent infection in neonates after the Norwood procedure: A retrospective chart review. Pediatr. Nephrol..

[19] Matejovic, M., et al., *Sepsis and acute kidney injury are bidirectional*, in *Controversies in Acute Kidney Injury*. 2011, Karger Publishers. p. 78–88. 10.1159/000329239 21921612

[20] Waters, M., et al., *CEBS—Chemical Effects in Biological Systems: a public data repository integrating study design and toxicity data with microarray and proteomics data.* Nucleic acids research, 2007. **36**(suppl_1): p. D892-D900. 10.1093/nar/gkm755 PMC2238989 17962311

[21] Sarwal M (2003). Molecular heterogeneity in acute renal allograft rejection identified by DNA microarray profiling. N. Engl. J. Med..

[22] Davis AP (2018). The comparative toxicogenomics database: Update 2019. Nucleic Acids Res..

[23] Goldstein SL (2013). Electronic health record identification of nephrotoxin exposure and associated acute kidney injury. Pediatrics.

[24] Lin K, Hu Y, Kong G (2019). Predicting in-hospital mortality of patients with acute kidney injury in the ICU using random forest model. Int. J. Med. Informatics.

[25] Su L-X (2011). Diagnostic value of urine sTREM-1 for sepsis and relevant acute kidney injuries: A prospective study. Crit. Care.

[26] DiMasi JA, Hansen RW, Grabowski HG (2003). The price of innovation: New estimates of drug development costs. J. Health Econ..

[27] Dickson M, Gagnon JP (2004). Key factors in the rising cost of new drug discovery and development. Nat. Rev. Drug Discov..

[28] Papapetropoulos A, Szabo C (2018). Inventing new therapies without reinventing the wheel: The power of drug repurposing. Br. J. Pharmacol..

[29] Wilde MI, Markham A (1996). Ondansetron. Drugs.

[30] Derkach V, Surprenant A, North R (1989). 5-HT3 receptors are membrane ion channels. Nature.

[31] Zheng GH (2018). MicroRNA-381-induced down-regulation of CXCR4 promotes the proliferation of renal tubular epithelial cells in rat models of renal ischemia reperfusion injury. J. Cell. Biochem..

[32] Kito, N., et al., *miRNA Profiles of Tubular Cells: Diagnosis of Kidney Injury.* BioMed Research International, 2015. **2015**. 10.1155/2015/465479 PMC4461729 26106607

[33] Aguado-Fraile E (2015). A pilot study identifying a set of microRNAs as precise diagnostic biomarkers of acute kidney injury. PLoS ONE.

[34] Si Y (2013). Dexmedetomidine protects against renal ischemia and reperfusion injury by inhibiting the JAK/STAT signaling activation. J. Transl. Med..

[35] Ozkok A (2016). NF-κB transcriptional inhibition ameliorates cisplatin-induced acute kidney injury (AKI). Toxicol. Lett..

[36] Nishtala PS, Chyou T (2020). Identifying drug combinations associated with acute kidney injury using association rules method. Drug Saf..

[37] Johnson AE (2016). MIMIC-III, a freely accessible critical care database. Sci. Data.

[38] Pollard TJ (2018). The eICU Collaborative Research Database, a freely available multi-center database for critical care research. Sci. Data.

[39] Nakamura K (2011). Sequence-specific error profile of Illumina sequencers. Nucleic Acids Res..

[40] Brand, M. *Incremental singular value decomposition of uncertain data with missing values*. in *European Conference on Computer Vision*. 2002. Springer.

[41] Rubin DB, Thomas N (1996). Matching using estimated propensity scores: Relating theory to practice. Biometrics.

[42] Stuart, E.A., et al. MatchIt: nonparametric preprocessing for parametric causal inference. *J. Stat. Software*, 2011.

[43] Famulski KS (2012). Molecular phenotypes of acute kidney injury in kidney transplants. J. Am. Soc. Nephrol..

[44] Team, R.C., *R: A language and environment for statistical computing.* 2013.

[45] Student, *The probable error of a mean.* Biometrika, 1908: p. 1–25.

[46] Mehta CR, Patel NR (1983). A network algorithm for performing Fisher's exact test in r× c contingency tables. J. Am. Stat. Assoc..

