# Supplementary material for: Effect of ondansetron on reducing ICU mortality in patients with acute kidney injury
Source: Sci Rep. 2021 Sep 30;11:19409. doi: 10.1038/s41598-021-98734-x (PMC8484575; doi:10.1038/s41598-021-98734-x)
Supplement: Supplementary file 1 — Supplementary Information. [file 41598_2021_98734_MOESM1_ESM.docx]

**Supplementary Materials:**

**Appendix A.** basic characteristics of MIMIC III patients

(Level 1 is for patients with the disease and 0 is for patients without the disease listed

For gender, 0 is for female patients and 1 is for male patients)

|  | level | 0 | 1 | p |
| --- | --- | --- | --- | --- |
| n |  | 5917 | 1110 |  |
| Heart rate (mean (SD)) |  | 87.29 (17.23) | 88.69 (17.52) | 0.013 |
| Systolic blood pressure (mean (SD)) |  | 115.51 (17.32) | 117.88 (17.75) | <0.001 |
| Diastolic blood pressure (mean (SD)) |  | 58.43 (10.98) | 60.91 (11.84) | <0.001 |
| Mean blood pressure (mean (SD)) |  | 75.10 (11.27) | 76.57 (12.03) | <0.001 |
| Respiratory (mean (SD)) |  | 20.21 (4.44) | 19.63 (4.15) | <0.001 |
| Temperature (mean (SD)) |  | 36.73 (0.74) | 36.78 (0.68) | 0.038 |
| oxygen saturation (mean (SD)) |  | 96.57 (3.47) | 96.77 (2.62) | 0.06 |
| Glucose (mean (SD)) |  | 147.33 (52.88) | 144.98 (49.30) | 0.171 |
| Congestive heart failure (%) | 0 | 5902 (99.7) | 1107 (99.7) | 1 |
|  | 1 | 15 (0.3) | 3 (0.3) |  |
| Pulmonary circulation (%) | 0 | 5869 (99.2) | 1098 (98.9) | 0.472 |
|  | 1 | 48 (0.8) | 12 (1.1) |  |
| Peripheral vascular (%) | 0 | 5753 (97.2) | 1090 (98.2) | 0.079 |
|  | 1 | 164 (2.8) | 20 (1.8) |  |
| Hypertension (%) | 0 | 5915 (100.0) | 1109 (99.9) | 0.967 |
|  | 1 | 2 (0.0) | 1 (0.1) |  |
| age (mean (SD)) |  | 89.07 (69.26) | 75.15 (54.41) | <0.001 |
| Anion gap (mean (SD)) |  | 16.08 (4.24) | 15.84 (3.97) | 0.08 |
| Albumin (mean (SD)) |  | 3.01 (0.50) | 3.08 (0.51) | <0.001 |
| Bicarbonate (mean (SD)) |  | 22.06 (5.09) | 21.92 (4.81) | 0.408 |
| Bilirubin (mean (SD)) |  | 2.48 (4.63) | 2.54 (4.97) | 0.707 |
| Creatinine (mean (SD)) |  | 2.03 (1.49) | 2.04 (1.66) | 0.849 |
| Hematocrit (mean (SD)) |  | 32.07 (5.59) | 31.74 (5.60) | 0.069 |
| Hemoglobin (mean (SD)) |  | 10.70 (1.93) | 10.60 (1.95) | 0.123 |
| Lactate (mean (SD)) |  | 2.89 (2.14) | 2.60 (1.58) | <0.001 |
| Platelet (mean (SD)) |  | 216.96 (121.20) | 220.49 (122.26) | 0.375 |
| Potassium (mean (SD)) |  | 4.34 (0.66) | 4.27 (0.66) | 0.002 |
| Sodium (mean (SD)) |  | 138.57 (5.82) | 137.61 (4.84) | <0.001 |
| Blood urea nitrogen (mean (SD)) |  | 42.30 (26.92) | 38.97 (27.34) | <0.001 |
| White blood cell (mean (SD)) |  | 13.26 (11.21) | 12.76 (10.11) | 0.166 |
| Stroke (%) | 0 | 5649 (95.5) | 1064 (95.9) | 0.624 |
|  | 1 | 268 (4.5) | 46 (4.1) |  |
| GENDER (%) | 0 | 2502 (42.3) | 534 (48.1) | <0.001 |
|  | 1 | 3415 (57.7) | 576 (51.9) |  |
| Surgical Status (%) | 0 | 3604 (60.9) | 558 (50.3) | <0.001 |
|  | 1 | 2313 (39.1) | 552 (49.7) |  |

**Appendix B.** Detailed ICU death rates among 7,027 patients with AKI with top 50 used drugs in MIMIC III database and 11,041 patients with AKI with top 50 used drugs in eICU database (NA: not available)

| **Drug name** | **Death rate in MIMIC III database (per 1000 patients)** | **Death rate in eICU database (per 1000 patients)** |
| --- | --- | --- |
| Acetaminophen | 183.73 | 123.83 |
| Albuterol | 261.03 | 174.80 |
| Amiodarone | 345.43 | 280.34 |
| Aspirin | 182.30 | 130.37 |
| Atorvastatin | 160.73 | 94.16 |
| Bisacodyl | 196.03 | 157.61 |
| Captopril | 134.40 | NA |
| Cefepime | 345.93 | 243.24 |
| Chlorhexidine | 330.19 | 228.55 |
| Dexamethasone | 377.64 | 58.44 |
| Diltiazem | 284.63 | 124.58 |
| Docusate | 180.91 | 136.16 |
| Famotidine | 265.08 | 183.45 |
| Fentanyl | 382.85 | 246.63 |
| Fluconazole | 332.65 | NA |
| Furosemide | 218.82 | 123.24 |
| Glucagon | 173.26 | 134.63 |
| Haloperidol | 165.63 | 136.31 |
| Heparin | 199.78 | 206.72 |
| Hydralazine | 214.61 | 144.30 |
| Hydromorphone | 161.32 | 83.55 |
| Insulin | 246.48 | 174.99 |
| Ipratropium | 263.69 | 173.37 |
| Lactulose | 332.63 | 248.77 |
| Levofloxacin | 273.95 | 146.83 |
| Levothyroxine | 220.18 | 109.68 |
| Lisinopril | 54.59 | 22.99 |
| Lorazepam | 290.84 | 225.53 |
| Magnesium Sulfate | 205.51 | 171.72 |
| Meropenem | 460.85 | 270.04 |
| Metoclopramide | 342.09 | 239.49 |
| Metoprolol | 253.65 | 160.90 |
| Metronidazole | 173.86 | 109.87 |
| Midazolam | 395.25 | 277.81 |
| Morphine | 382.12 | 238.65 |
| Nitroglycerin | 201.01 | 76.45 |
| Norepinephrine | 505.18 | 342.60 |
| Ondansetron | 144.14 | 125.33 |
| Oxycodone | 94.86 | 56.86 |
| Pantoprazole | 258.62 | 153.88 |
| Phytonadione | 368.24 | 333.33 |
| Piperacillin | 340.94 | 210.58 |
| Prednisone | 199.69 | 63.38 |
| Propofol | 292.47 | 266.52 |
| Senna | 179.30 | 207.39 |
| Tacrolimus | 169.81 | 54.55 |
| Vancomycin | 304.06 | 236.32 |
| Warfarin | 67.78 | 40.49 |
| All | 231.68 | 152.98 |

**Appendix C.** Detailed information of medications identified with significant effects on Intensive Care Unit mortality for Patients with Acute Kidney Injury (Y, yes with clinical trials and/or literature supports, N, no report)

| **Medication** | **Reducing ICU mortality**  **of Patients with AKI by logistic regression analysis** | **Reported protective effect in all ICU patients** | **Reported protective effect for Patients with AKI in ICU** | **Medical use of drug** |
| --- | --- | --- | --- | --- |
| Warfarin | Y | Y[1] | N | treat and prevent blood clots |
| Oxycodone | Y | Y[2] | N | treat moderate to severe pain |
| Heparin | Y | Y[3] | N | anticoagulant, also used to treat heart attacks and unstable angina |
| Magnesium Sulfate | Y | Y[4] | Y[5] | prevention and control of seizures in preeclampsia and eclampsia |
| Haloperidol | Y | N | N | antipsychotic[6] |
| Glucagon | Y | Y[7] | N | treat severe low blood sugar |
| Metoprolol | Y | Y[8] | N | beta blocker |
| Ondansetron | Y | N | N | prevent nausea and vomiting[9] |
| Furosemide | Y | Y[10] | Y[11] | treat fluid retention (edema) and swelling |
| Acetaminophen | Y | N | N | analgesic[12] |
| Hydralazine | Y | Y[13] | Y[14] | treat high blood pressure |
| Hydromorphone | Y | N | N | treat moderate to severe pain[15] |
| Docusate | Y | N | N | treat constipation[16] |
| Pantoprazole | Y | N | N | treatment of stomach ulcers, erosive esophagitis due to gastroesophageal reflux disease (GERD)[17] |
| Midazolam | N | Y[18] | N | used for anesthesia, procedural sedation, trouble sleeping, and severe agitation |
| Lorazepam | N | N | N | treat anxiety disorders, trouble sleeping, active seizures, and chemotherapy-induced nausea and vomiting[19] |
| Meropenem | N | Y[20] | N | Antibiotics |
| Fentanyl | N | Y[21] | N | narcotic, treat severe pain |
| Amiodarone | N | N | N | treat heart rhythm problems[22] |
| Norepinephrine | N | Y[23] | N | treat low blood pressure and heart failure |
| Morphine | N | Y[1] | N | treat moderate to severe pain |

**Appendix D.** The detailed basic characteristics of eICU patients before matching

(Level 1 is for patients with the disease and 0 is for patients without the disease listed)

|  | level | 0 | 1 | p |
| --- | --- | --- | --- | --- |
| n |  | 7602 | 3439 |  |
| Gender (%) | Female | 3312 (43.6) | 1525 (44.3) | 0.599 |
|  | Male | 4289 (56.4) | 1914 (55.7) |  |
|  | Unknown | 1 (0.0) | 0 (0.0) |  |
| Age (%) | <=49 | 1065 (14.0) | 564 (16.4) | <0.001 |
|  | >49 | 1265 (16.6) | 645 (18.8) |  |
|  | >59 | 1727 (22.7) | 767 (22.3) |  |
|  | >69 | 1736 (22.8) | 794 (23.1) |  |
|  | >79 | 1452 (19.1) | 535 (15.6) |  |
|  | >89 | 357 (4.7) | 134 (3.9) |  |
| Congestive heart failure (%) | 0 | 6386 (84.0) | 2978 (86.6) | <0.001 |
|  | 1 | 1216 (16.0) | 461 (13.4) |  |
| Cardiac arrhythmias (%) | 0 | 5735 (75.4) | 2645 (76.9) | 0.099 |
|  | 1 | 1867 (24.6) | 794 (23.1) |  |
| Hypertension (%) | 0 | 6219 (81.8) | 2846 (82.8) | 0.239 |
|  | 1 | 1383 (18.2) | 593 (17.2) |  |
| Hypothyroidism (%) | 0 | 7224 (95.0) | 3315 (96.4) | 0.002 |
|  | 1 | 378 (5.0) | 124 (3.6) |  |
| Coagulopathy (%) | 0 | 7043 (92.6) | 3236 (94.1) | 0.006 |
|  | 1 | 559 (7.4) | 203 (5.9) |  |
| Electrolyte disorder (%) | 0 | 5595 (73.6) | 2605 (75.7) | 0.018 |
|  | 1 | 2007 (26.4) | 834 (24.3) |  |
| Diabetes (%) | 0 | 6020 (79.2) | 2856 (83.0) | <0.001 |
|  | 1 | 1582 (20.8) | 583 (17.0) |  |
| Liver disease (%) | 0 | 6866 (90.3) | 3122 (90.8) | 0.463 |
|  | 1 | 736 (9.7) | 317 (9.2) |  |
| COPD (%) | 0 | 6779 (89.2) | 3166 (92.1) | <0.001 |
|  | 1 | 823 (10.8) | 273 (7.9) |  |
| Tumor (%) | 0 | 7220 (95.0) | 3232 (94.0) | 0.035 |
|  | 1 | 382 (5.0) | 207 (6.0) |  |
| Respiratory failure (%) | 0 | 4284 (56.4) | 2184 (63.5) | <0.001 |
|  | 1 | 3318 (43.6) | 1255 (36.5) |  |
| Surgical Status (%) | 0 | 6833 (89.9) | 2888 (84.0) | <0.001 |
|  | 1 | 769 (10.1) | 551 (16.0) |  |
| Temperature (mean (SD)) |  | 36.28 (1.29) | 36.31 (1.18) | 0.182 |
| Respiratory rate (mean (SD)) |  | 26.62 (14.29) | 27.74 (15.16) | <0.001 |
| Heart rate (mean (SD)) |  | 105.88 (31.38) | 105.74 (31.19) | 0.82 |
| Mean blood pressure (mean (SD)) |  | 81.65 (45.15) | 82.56 (45.17) | 0.324 |
| Creatinine (mean (SD)) |  | 2.73 (2.21) | 2.85 (2.40) | 0.012 |

COPD: Chronic obstructive pulmonary disease

**Appendix E.** The detailed basic characteristics of eICU patients after matching

(Level 1 is for patients with the disease and 0 is for patients without the disease listed)

|  | level | 0 | 1 | p |
| --- | --- | --- | --- | --- |
| n |  | 3423 | 3423 |  |
| Gender (%) | Female | 1490 (43.5) | 1516 (44.3) | 0.543 |
|  | Male | 1933 (56.5) | 1907 (55.7) |  |
| Age (%) | <=49 | 568 (16.6) | 561 (16.4) | 0.965 |
|  | >49 | 645 (18.8) | 641 (18.7) |  |
|  | >59 | 768 (22.4) | 765 (22.3) |  |
|  | >69 | 773 (22.6) | 789 (23.0) |  |
|  | >79 | 547 (16.0) | 533 (15.6) |  |
|  | >89 | 122 (3.6) | 134 (3.9) |  |
| Congestive heart failure (%) | 0 | 2976 (86.9) | 2963 (86.6) | 0.669 |
|  | 1 | 447 (13.1) | 460 (13.4) |  |
| Cardiac arrhythmias (%) | 0 | 2656 (77.6) | 2633 (76.9) | 0.526 |
|  | 1 | 767 (22.4) | 790 (23.1) |  |
| Hypertension (%) | 0 | 2820 (82.4) | 2834 (82.8) | 0.679 |
|  | 1 | 603 (17.6) | 589 (17.2) |  |
| Hypothyroidism (%) | 0 | 3286 (96.0) | 3301 (96.4) | 0.375 |
|  | 1 | 137 (4.0) | 122 (3.6) |  |
| Coagulopathy (%) | 0 | 3220 (94.1) | 3221 (94.1) | 1 |
|  | 1 | 203 (5.9) | 202 (5.9) |  |
| Electrolyte disorder (%) | 0 | 2583 (75.5) | 2593 (75.8) | 0.8 |
|  | 1 | 840 (24.5) | 830 (24.2) |  |
| Diabetes (%) | 0 | 2851 (83.3) | 2842 (83.0) | 0.796 |
|  | 1 | 572 (16.7) | 581 (17.0) |  |
| Liver disease (%) | 0 | 3100 (90.6) | 3108 (90.8) | 0.771 |
|  | 1 | 323 (9.4) | 315 (9.2) |  |
| COPD (%) | 0 | 3164 (92.4) | 3152 (92.1) | 0.619 |
|  | 1 | 259 (7.6) | 271 (7.9) |  |
| Tumor (%) | 0 | 3214 (93.9) | 3217 (94.0) | 0.919 |
|  | 1 | 209 (6.1) | 206 (6.0) |  |
| Respiratory failure (%) | 0 | 2149 (62.8) | 2171 (63.4) | 0.599 |
|  | 1 | 1274 (37.2) | 1252 (36.6) |  |
| Surgical Status (%) | 0 | 2892 (84.5) | 2886 (84.3) | 0.868 |
|  | 1 | 531 (15.5) | 537 (15.7) |  |
| Temperature (mean (SD)) |  | 36.34 (1.21) | 36.31 (1.18) | 0.241 |
| Respiratory rate (mean (SD)) |  | 27.94 (14.40) | 27.71 (15.15) | 0.528 |
| Heart rate (mean (SD)) |  | 105.40 (30.80) | 105.68 (31.14) | 0.711 |
| Mean blood pressure (mean (SD)) |  | 82.84 (45.48) | 82.60 (45.19) | 0.828 |
| Creatinine (mean (SD)) |  | 2.84 (2.39) | 2.85 (2.40) | 0.88 |

COPD: Chronic obstructive pulmonary disease

**Appendix F.** Enriched pathways in the Ondansetron and AKI biosets

| **Pathways** | **Genes** | **Bitset1**  **(Ondansetron)** | | **Bioset2 (AKI)** | **Bioset3 (AKI)** | **Bioset4(AKI)** |
| --- | --- | --- | --- | --- | --- | --- |
| Pathways in cancer | 311 | | 32(2.1E-17) | 66(5.6E-30) | 89(5.8E-40) | 82(5.7E-36) |
| Predicted Gene Targets for miR-381 | 472 | | 42(4.3E-20) | 83(2.2E-30) | 103(1.9E-34) | 87(6.2E-26) |
| Predicted Gene Targets for miR-200b | 428 | | 34(6.8E-15) | 75(2.9E-27) | 101(1.1E-36) | 86(1.7E-28) |
| Positive regulation of cell differentiation | 482 | | 39(2.7E-17) | 67(7.8E-21) | 89(4.2E-28) | 92(4.7E-31) |
| Predicted Gene Targets for miR-101 | 386 | | 24(1.7E-8) | 69(3.0E-26) | 94(5.0E-36) | 75(8.3E-24) |
| Kinase binding | 426 | | 41(3.7E-21) | 56(4.7E-18) | 77(7.7E-26) | 79(2.7E-28) |
| Targets of MicroRNA CAGTATT,MIR-200B,MIR-200C,MIR-429 | 448 | | 32(1.1E-12) | 77(4.1E-27) | 93(1.4E-29) | 80(1.3E-23) |
| Predicted Gene Targets for miR-26 | 448 | | 36(8.6E-16) | 71(1.5E-23) | 93(3.1E-29) | 83(3.2E-24) |
| Response to peptide hormone stimulus | 326 | | 31(5.6E-17) | 59(5.1E-22) | 86(5.2E-33) | 65(2.0E-20) |
| Genes involved in Hemostasis | 420 | | 42(4.3E-21) | 73(3.6E-26) | 80(1.5E-22) | 77(1.5E-21) |

**Appendix G.** Detailed gene expression fold changes in ondansetron and AKI biosets

| **Gene** | **Bioset1 (Ondansetron)** | **Bioset2 (AKI)** | **Bioset3 (AKI)** | **Bioset4 (AKI)** |
| --- | --- | --- | --- | --- |
| Rela | -265 | 1.37 | - | 1.64 |
| Jak1 | -10.7 | 1.74 | 0.65 | 0.36 |
| Ctbp2 | -10.3 | - | - | - |
| Fn1 | -10.3 | - | - | - |
| Rb1 | -9.02 | - | 1.51 | 1.48 |
| Ctnnb1 | -6.88 | -2.1 | -2.1 | -2.42 |
| Pld1 | -5.95 | - | 1.52 | 1.67 |
| Mapk9 | -5.48 | 1.29 | -1.8 | - |
| Pten | -4.92 | 1.86 | 1.67 | 0.03 |
| Casp3 | -4.18 | 1.44 | -1.53 | -1.93 |
| Mapk1 | -4.09 | 2.94 | 1.88 | 1.89 |
| Lamb2 | -4.03 | - | - | - |
| Tgfa | -3.86 | -1.47 | 1.49 | - |
| Cdkn2b | -3.79 | - | - | - |
| Pik3r1 | -3.77 | 1.91 | - | - |
| Prkcb | -3.73 | 0.62 | -0.31 | -0.49 |
| Itgb1 | -3.18 | - | - | - |
| Ctnna1 | -2.95 | 1.63 | 2.1 | 1.96 |
| Birc2 | -2.85 | - | - | - |
| Crk | -2.78 | - | - | - |
| Kras | -2.58 | 1.86 | 1.61 | - |
| Smo | -2.45 | - | - | 1.23 |
| Met | -2.32 | - | 1.81 | 1.94 |
| Msh2 | -2.29 | - | - | - |
| Erbb2 | -2.24 | - | 1.64 | 1.73 |
| Cycs | -2.08 | 1.78 | - | - |
| Mapk3 | -1.98 | - | - | - |
| Tpr | -1.93 | 1.81 | 0.29 | 1.64 |
| Mtor | -1.75 | 1.33 | - | - |
| Cdc42 | -1.7 | -2.25 | -1.91 | -2.21 |
| Plcg2 | -1.63 | 1.98 | -1.41 | -1.46 |
| Tgfb1 | -1.54 | -2.17 | -1.37 | -1.44 |
| Mecom | 1.46 | 2.08 | - | 1.76 |
| Pdgfrb | 1.49 | - | - | 1.65 |
| Raf1 | 1.51 | -0.08 | -0.03 | -0.12 |
| Hdac1 | 1.52 | - | - | - |
| Tgfb3 | 1.83 | - | - | - |
| Axin2 | 2.12 | - | - | - |
| Gsk3b | 2.3 | - | 1.46 | 1.87 |
| Sos2 | 2.47 | -1.39 | - | - |

‘-’: means that the gene expression is within the fold change range of -1.2 to 1.2.

**Appendix H.** Variables extracted from MIMIC III database for logistic regression

| **First-day Vital and lab test Information/comorbidities** | **Medication Use Information** | **Other Variables** |
| --- | --- | --- |
| Mean oxygen saturation | Morphine | SOFA |
| Mean temperature | Norepinephrine | SAPS-II |
| Mean respiration rate | Warfarin | Elixhauser's Comorbidity Index |
| Mean potassium level | Magnesium Sulfate | KDIGO stages in the first 48 hours |
| Mean white blood cell count | Fluconazole | Surgical status |
| Mean prothrombin time | Furosemide |  |
| Mean hematocrit level | Oxycodone |  |
| Mean creatinine level | Haloperidol |  |
| Mean hemoglobin level | Heparin |  |
| Mean platelet level | Metoclopramide |  |
| peripheral vascularity | Metoprolol |  |
| Mean blood pressure | Meropenem |  |
| Mean partial thromboplastin time | Lisinopril |  |
| Mean lactic acid level | Propofol |  |
| Mean diastolic blood pressure | Captopril |  |
| Mean chloride level | Amiodarone |  |
| Mean sodium level | Hydralazine |  |
| Mean bicarbonate level | Acetaminophen |  |
| Mean albumin level | Piperacillin |  |
| Mean systolic blood pressure | Phytonadione |  |
| Mean Glucose level | Lactulose |  |
| Mean bands cell count | Ondansetron |  |
| Pulmonary circulation | Nitroglycerin |  |
| Mean level of bilirubin | Fentanyl |  |
| Mean interaction between the Cardiac Rapidly | Albuterol |  |
| Mean heart rate | Ipratropium |  |
| Mean anion gap_ | Bisacodyl |  |
| Mean blood urea nitrogen | Tacrolimus |  |
| Coagulopathy | Prednisone |  |
| Liver disease | Senna |  |
| Metastatic cancer | Aspirin |  |
| Stroke | Cefepime |  |
| Chronic pulmonary | Hydromorphone |  |
| Lymphoma | Insulin |  |
| Alcohol abuse | Levofloxacin |  |
| Rheumatoid arthritis | Metronidazole |  |
| Renal failure | Lorazepam |  |
| Hypertension | Famotidine |  |
| Congestive heart failure | Midazolam |  |
| Solid tumor | Chlorhexidine |  |
| Gender | Dexamethasone |  |
| Age | Diltiazem |  |
|  | Glucagon |  |
|  | Pantoprazole |  |
|  | Docusate |  |
|  | Vancomycin |  |
|  | Atorvastatin |  |
|  | Levothyroxine |  |

SOFA Score: Sequential Organ Failure Assessment Score

SAPSII: Simplified Acute Physiology Score (SAPS) II

KDIGO: Kidney Disease Improving Global Outcomes

**Appendix I.** ICD9 codes for comorbidities (from <https://github.com/MIT-LCP/mimic-code/blob/52d7df53348a6e25dfbe795c0e28c389efc40be9/mimic-iii/concepts/comorbidity/elixhauser_quan.sql>)

| **Disease** | **ICD9 codes** |
| --- | --- |
| Congestive heart failure | when icd9_code in ('39891','40201','40211','40291','40401','40403','40411','40413','40491','40493') |
|  | when SUBSTR(icd9_code, 1, 4) in ('4254','4255','4257','4258','4259') |
|  | when SUBSTR(icd9_code, 1, 3) in ('428') |
| Cardiac arrhythmias | when icd9_code in ('42613','42610','42612','99601','99604') |
|  | when SUBSTR(icd9_code, 1, 4) in ('4260','4267','4269','4270','4271','4272','4273','4274','4276','4278','4279','7850','V450','V533') |
| Valvular disease | when SUBSTR(icd9_code, 1, 4) in ('0932','7463','7464','7465','7466','V422','V433') |
|  | when SUBSTR(icd9_code, 1, 3) in ('394','395','396','397','424') |
| Pulmonary circulation disorder | when SUBSTR(icd9_code, 1, 4) in ('4150','4151','4170','4178','4179') |
|  | when SUBSTR(icd9_code, 1, 3) in ('416') |
| Peripheral vascular disorder | when SUBSTR(icd9_code, 1, 4) in ('0930','4373','4431','4432','4438','4439','4471','5571','5579','V434') |
|  | when SUBSTR(icd9_code, 1, 3) in ('440','441') |
| hypertension | when SUBSTR(icd9_code, 1, 3) in ('401') |
|  | when SUBSTR(icd9_code, 1, 3) in ('402','403','404','405') |
| Paralysis | when SUBSTR(icd9_code, 1, 4) in ('3341','3440','3441','3442','3443','3444','3445','3446','3449') |
|  | when SUBSTR(icd9_code, 1, 3) in ('342','343') |
| Other neurological | when icd9_code in ('33392') |
|  | when SUBSTR(icd9_code, 1, 4) in ('3319','3320','3321','3334','3335','3362','3481','3483','7803','7843') |
|  | when SUBSTR(icd9_code, 1, 3) in ('334','335','340','341','345') |
| Chronic pulmonary disease | when SUBSTR(icd9_code, 1, 4) in ('4168','4169','5064','5081','5088') |
|  | when SUBSTR(icd9_code, 1, 3) in ('490','491','492','493','494','495','496','500','501','502','503','504','505') |
| Diabetes | when SUBSTR(icd9_code, 1, 4) in ('2500','2501','2502','2503')' |
|  | when SUBSTR(icd9_code, 1, 4) in ('2504','2505','2506','2507','2508','2509') |
| Hypothyroidism | when SUBSTR(icd9_code, 1, 4) in ('2409','2461','2468') |
|  | when SUBSTR(icd9_code, 1, 3) in ('243','244') |
| Renal failure | when icd9_code in ('40301','40311','40391','40402','40403','40412','40413','40492','40493') |
|  | when SUBSTR(icd9_code, 1, 4) in ('5880','V420','V451') |
|  | when SUBSTR(icd9_code, 1, 3) in ('585','586','V56') |
| Liver disease | when icd9_code in ('07022','07023','07032','07033','07044','07054') |
|  | when SUBSTR(icd9_code, 1, 4) in ('0706','0709','4560','4561','4562','5722','5723','5724','5728','5733','5734','5738','5739','V427') |
|  | when SUBSTR(icd9_code, 1, 3) in ('570','571') |
| ulcer | when SUBSTR(icd9_code, 1, 4) in ('5317','5319','5327','5329','5337','5339','5347','5349') |
| HIV and AIDS | when SUBSTR(icd9_code, 1, 3) in ('042','043','044') |
| Lymphoma | when SUBSTR(icd9_code, 1, 4) in ('2030','2386') |
|  | when SUBSTR(icd9_code, 1, 3) in ('200','201','202') |
| Metastatic cancer | when SUBSTR(icd9_code, 1, 3) in ('196','197','198','199') |
| Tumor | when SUBSTR(icd9_code, 1, 3) in |
|  | ( |
|  | '140','141','142','143','144','145','146','147','148','149','150','151','152' |
|  | ,'153','154','155','156','157','158','159','160','161','162','163','164','165' |
|  | ,'166','167','168','169','170','171','172','174','175','176','177','178','179' |
|  | ,'180','181','182','183','184','185','186','187','188','189','190','191','192' |
|  | ,'193','194','195' |
|  | ) |
| Rheumatoid arthritis | when icd9_code in ('72889','72930') |
|  | when SUBSTR(icd9_code, 1, 4) in ('7010','7100','7101','7102','7103','7104','7108','7109','7112','7193','7285') |
|  | when SUBSTR(icd9_code, 1, 3) in ('446','714','720','725') |
| Coagulation deficiency | when SUBSTR(icd9_code, 1, 4) in ('2871','2873','2874','2875') |
|  | when SUBSTR(icd9_code, 1, 3) in ('286') |
| Obesity | when SUBSTR(icd9_code, 1, 4) in ('2780') |
| Weight loss | when SUBSTR(icd9_code, 1, 4) in ('7832','7994') |
|  | when SUBSTR(icd9_code, 1, 3) in ('260','261','262','263') |
| Fluid and electrolyte disorders | when SUBSTR(icd9_code, 1, 4) in ('2536') |
|  | when SUBSTR(icd9_code, 1, 3) in ('276') |
| anemia | when SUBSTR(icd9_code, 1, 4) in ('2800') |
|  | when SUBSTR(icd9_code, 1, 4) in ('2801','2808','2809') |
|  | when SUBSTR(icd9_code, 1, 3) in ('281') |
| Alcohol abuse | when SUBSTR(icd9_code, 1, 4) in ('2652','2911','2912','2913','2915','2918','2919','3030','3039','3050','3575','4255','5353','5710','5711','5712','5713','V113') |
|  | when SUBSTR(icd9_code, 1, 3) in ('980') |
| Drug abuse | when icd9_code in ('V6542') |
|  | when SUBSTR(icd9_code, 1, 4) in ('3052','3053','3054','3055','3056','3057','3058','3059') |
|  | when SUBSTR(icd9_code, 1, 3) in ('292','304') |
| Psychoses | when icd9_code in ('29604','29614','29644','29654') |
|  | when SUBSTR(icd9_code, 1, 4) in ('2938') |
|  | when SUBSTR(icd9_code, 1, 3) in ('295','297','298') |
| Depression | when SUBSTR(icd9_code, 1, 4) in ('2962','2963','2965','3004') |
|  | when SUBSTR(icd9_code, 1, 3) in ('309','311') |

1. Beard Jr, E.L., *The american society of health system pharmacists.* JONA'S healthcare law, ethics and regulation, 2001. **3**(3): p. 78-79.

2. Moradi, M., et al., *Use of oxycodone in pain management.* Anesthesiology and pain medicine, 2012. **1**(4): p. 262.

3. Agnelli, G., et al., *Enoxaparin plus compression stockings compared with compression stockings alone in the prevention of venous thromboembolism after elective neurosurgery.* New England Journal of Medicine, 1998. **339**(2): p. 80-85.

4. Pryde, P.G. and R. Mittendorf, *Contemporary usage of obstetric magnesium sulfate: indication, contraindication, and relevance of dose.* Obstetrics & Gynecology, 2009. **114**(3): p. 669-673.

5. Firouzi, A., et al., *Intravenous magnesium sulfate: new method in prevention of contrast-induced nephropathy in primary percutaneous coronary intervention.* International urology and nephrology, 2015. **47**(3): p. 521-525.

6. Tyler, M.W., J. Zaldivar-Diez, and S.J. Haggarty, *Classics in Chemical Neuroscience: Haloperidol.* ACS Chem Neurosci, 2017. **8**(3): p. 444-453.

7. Farivar, M., et al., *Effect of insulin and glucagon on fulminant murine hepatitis.* New England Journal of Medicine, 1976. **295**(27): p. 1517-1519.

8. Mohan, J.C., et al., *Rediscovering Chirality - Role of S-Metoprolol in Cardiovascular Disease Management.* J Assoc Physicians India, 2017. **65**(6): p. 74-79Jagdish.

9. Roila, F. and A. Del Favero, *Ondansetron clinical pharmacokinetics.* Clin Pharmacokinet, 1995. **29**(2): p. 95-109.

10. Buggey, J., et al., *A reappraisal of loop diuretic choice in heart failure patients.* American heart journal, 2015. **169**(3): p. 323-333.

11. Phakdeekitcharoen, B. and K. Boonyawat, *The added-up albumin enhances the diuretic effect of furosemide in patients with hypoalbuminemic chronic kidney disease: a randomized controlled study.* BMC nephrology, 2012. **13**(1): p. 92.

12. Aminoshariae, A. and A. Khan, *Acetaminophen: old drug, new issues.* J Endod, 2015. **41**(5): p. 588-93.

13. Howland, R.D., et al., *Lippincott's illustrated reviews: Pharmacology*. 2006: Lippincott Williams & Wilkins Philadelphia.

14. Tampe, B., et al., *Low-dose hydralazine prevents fibrosis in a murine model of acute kidney injury–to–chronic kidney disease progression.* Kidney international, 2017. **91**(1): p. 157-176.

15. Murray, A. and N.A. Hagen, *Hydromorphone.* Journal of pain and symptom management, 2005. **29**(5): p. 57-66.

16. Tarumi, Y., et al., *Randomized, double-blind, placebo-controlled trial of oral docusate in the management of constipation in hospice patients.* Journal of pain and symptom management, 2013. **45**(1): p. 2-13.

17. Cheer, S.M., et al., *Pantoprazole.* Drugs, 2003. **63**(1): p. 101-132.

18. Ozdemir, D., et al., *Efficacy of continuous midazolam infusion and mortality in childhood refractory generalized convulsive status epilepticus.* Seizure, 2005. **14**(2): p. 129-132.

19. Bush, G., et al., *Catatonia. II. Treatment with lorazepam and electroconvulsive therapy.* Acta Psychiatrica Scandinavica, 1996. **93**(2): p. 137-143.

20. Cheng, A.C., et al., *Outcomes of patients with melioidosis treated with meropenem.* Antimicrobial agents and chemotherapy, 2004. **48**(5): p. 1763-1765.

21. Goodman, L.S., *Goodman and Gilman's the pharmacological basis of therapeutics*. Vol. 1549. 1996: McGraw-Hill New York.

22. Mullord, P. and A. Sargent, *Pharmacological conversion of AV nodal re-entry tachycardia with adenosine.* British Journal of Cardiac Nursing, 2011. **6**(4): p. 178-183.

23. Rang, H., et al., *Chapter 14: Noradrenergic transmission.* Rang & Dale's Pharmacology. Elsevier Health Sciences, 2014: p. 177-196.
